# A child with Gradenigo syndrome presenting with meningism: a case report

**DOI:** 10.1186/s12887-019-1754-6

**Published:** 2019-10-13

**Authors:** A. S. Athapathu, E. R. S. Bandara, A. A. H. S. Aruppala, K. M. A. U. Chandrapala, Sachith Mettananda

**Affiliations:** 1 University Paediatrics Unit, Colombo North Teaching Hospital, Ragama, Sri Lanka; 2 Department of Paediatrics, Faculty of Medicine, University of Kelaniya, Ragama, Sri Lanka

**Keywords:** Gradenigo syndrome, Abducens nerve palsy, Fever, Headache, Photophobia

## Abstract

**Background:**

The symptoms of meningitis which include fever, headache, photophobia and irritability along with abducens nerve palsy pose a diagnostic dilemma requiring urgent attention. Here we report how such a dilemma was methodically and sequentially resolved using anatomical knowledge supported by neuroimaging and the eventual diagnosis of Gradenigo syndrome was made.

**Case presentation:**

A 6-year-old previously healthy boy from Sri Lanka presented with high grade fever, headache, photophobia and left eye pain for 10 days and diplopia for 2 days duration. Neurological examination was unremarkable except for left sided abducens nerve palsy. He had high inflammatory markers and white blood cell count. A tentative differential diagnosis of acute bacterial meningitis complicated by cerebral oedema, acute hydrocephalus or cerebral abscess was made. However, non-contrast CT brain, cerebrospinal fluid analysis and electroencephalogram were normal leading to a diagnostic dilemma. MRI brain with contrast performed 3 days later due to limited resources revealed left mastoiditis extending to petrous temporal bone confirming Gradenigo syndrome.

**Conclusion:**

This case report highlights the importance of a thorough physical examination in children presenting with unrelated neurological symptoms and signs. Unilateral abducens nerve palsy raises the suspicion of increased intracranial pressure and neuroimaging is vital in diagnostic uncertainties. Gradenigo syndrome emphasises the importance of incorporating anatomical knowledge into clinical practice.

## Background

Fever, headache, photophobia and irritability suggest meningitis in a child. When these symptoms are combined with unilateral abducens nerve palsy a diagnostic dilemma which requires urgent attention arises. Here, we describe a six-year old child with fever, headache, photophobia and abducens nerve palsy who was eventually diagnosed as Gradenigo Syndrome.

## Case presentation

A six-year old previously healthy Sri Lankan boy presented with high grade fever (> 101 °F) for 10 days duration. He also complained of headache, left eye pain and photophobia for the same duration and double vision for 2 days. He did not have nausea, vomiting, malaise or seizures. There was no ear pain or hearing loss. He had been taking oral cefuroxime prescribed by a general practitioner for the febrile illness during previous 5 days.

**Fig. 1:**
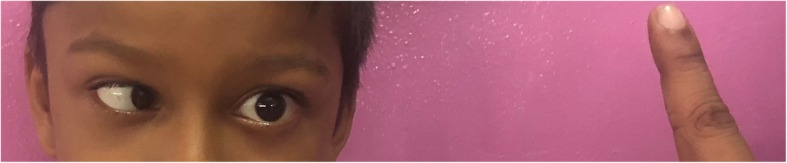
A photograph of the child demonstrating impaired lateral movement of the left eye due to left abducens nerve palsy

On examination he was ill looking, irritable and febrile. There was no neck stiffness and the Kernig sign was negative. There were no rashes. Examination of cranial nerves revealed left abducens nerve palsy (Fig. 1). Examination of visual fields, pupillary light reflexes, optic fundi, other cranial nerves and rest of the neurological examination were normal. Cardiovascular system examination including pulse rate and blood pressure was normal. The tympanic membranes could not be visualized immediately due to the presence of ear wax which could not be removed on the day of presentation due to limitation of resources. There was no tenderness or erythema over the mastoid process. Initial investigations results revealed; C-reactive protein – 161 mg/dL, haemoglobin – 11 g/dL, white blood cell count – 11,000/μL (Neutrophils 75%) and platelet count – 456,000/μL.

**Fig. 2:**
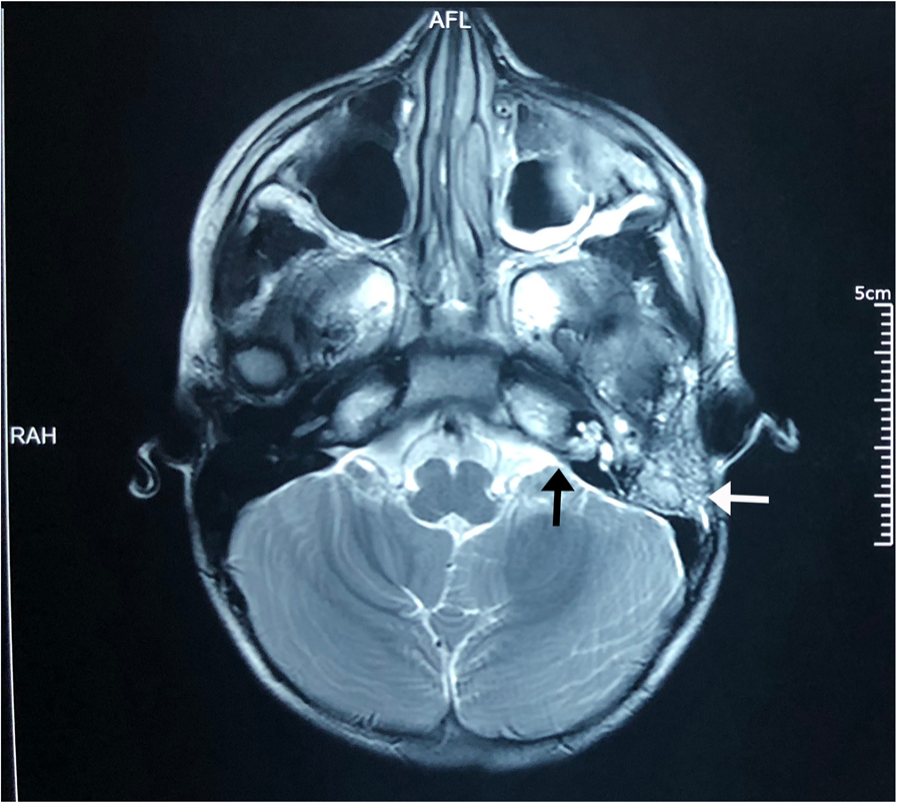
Contrast enhanced MRI brain demonstrating left mastoiditis; white arrow points to the mastoid sinus with mastoiditis and black arrow points to the petrous bone

A tentative differential diagnosis of acute bacterial meningitis complicated by cerebral oedema, acute hydrocephalus or cerebral abscess was made. The child was commenced on intravenous cefotaxime, intravenous aciclovir and 3% sodium chloride after obtaining blood for bacterial culture. Urgent non-contrast computed tomography (CT) brain was performed due to limited resources and was however reported as normal with no evidence of cerebral oedema, hydrocephalus or intracranial abscesses. Lumbar puncture and electroencephalogram which were done on the following day were normal with normal cerebrospinal (CSF) opening pressure. Both blood and CSF cultures showed no bacterial growth. To resolve the diagnostic dilemma Magnetic Resonance Image (MRI) brain with contrast was ordered but could not be performed until 3 days after admission due to limitations in resources. MRI brain revealed left mastoiditis extending up to the petrous temporal bone confirming the diagnosis of Gradenigo syndrome (Fig. 2). There were no cavernous sinus or other cerebral sinus thromboses. Tympanometry revealed type B pattern in left ear consistent with a middle ear effusion.

The antimicrobial therapy was changed to intravenous vancomycin and ceftazidime and continued for 14 days. Tympanostomy and Grommet insertion were done. Fever settled after starting appropriate antibiotics and the child made a gradual recovery with improvement in eye movements and inflammatory markers. The child was discharged on oral co-amoxiclav and ciprofloxacin for further period of 4 weeks.

## Discussion and conclusions

Gradenigo syndrome describes the triad of petrositis, unilateral abducens nerve palsy and pain in the distribution of the trigeminal nerve [1]. It serves as a classic example where the clinical features can be vividly described based on anatomy [2]. However, as in this case, it can lead to a diagnostic dilemma. Unawareness of the condition and failure of proper clinical evaluation could result in delayed diagnosis and life-threatening consequences [3–5].

When a child presents with fever, headache and photophobia, central nervous system infections should be excluded. Nevertheless, when these symptoms are associated with unilateral abducens nerve palsy it is clinically alarming especially in a setting of limited availability of neuroimaging. In our patient normal non-contrast CT brain was reassuring however, could not identify the cause for abducens nerve palsy. Ultimate diagnosis was made by contrast enhanced MRI. Headache in our child was most likely due to mastoiditis and facial pain in the distribution of the trigeminal nerve. Mastoiditis with absent clinical findings such as tenderness over the mastoid process has been described in literature [6]. The complaint of photophobia could be due to difficulties perceived due to diplopia and paralytic squint in abducens nerve palsy. Therefore, the clinical diagnosis was difficult to establish.

Other differential diagnoses with a similar presentation are cerebral sino-venous thrombosis, cavernous sinus thrombosis and acute demyelinating encephalomyelitis [7]. Additionally, infections like typhus, herpes simplex virus and meningitis are known to be associated with abducens nerve palsy [8]. Meningitis, otitis media or mastoiditis itself can predispose to cerebral sino-venous thrombosis. Non-contrast CT is very insensitive for the above diagnoses. Although MRI brain did not reveal any thromboses, ideally contrast CT venography or Magnetic Resonance Venography should have been performed to exclude these conditions. In the event of suspicion about cerebral sinus thrombosis, anticoagulation with either unfractionated heparin or low molecular weight heparin would be beneficial.

In Gradenigo syndrome, infection in the middle ear spreads to mastoid air cells causing mastoiditis and petrous temporal bones resulting in petrositis [3]. The trigeminal ganglion is located near the apex of the petrous temporal bone in a cavity called the Meckel’s cave. Inflammation of the ophthalmic division of the trigeminal nerve leads to retro-orbital pain. The abducens nerve passes through Dorello’s canal, which is bounded medially by the clinoid process, laterally by the sphenoidal ridge and posterior-superiorly by the petro-sphenoidal Gruber’s ligament. Petrositis can directly extend to compress the abducens nerve at this narrow canal, leading to lateral rectus palsy resulting in diplopia. Additionally, facial nerve could also be affected due to acute otitis media with intra-temporal extension of the infection. Extension to the base of the skull with involvement of ninth, tenth and eleventh cranial nerves is termed Vernet syndrome.

Traditional management of Gradenigo syndrome had been surgical, with mastoidectomy and decompression of the petrous apex [9]. However emerging evidence suggests medical management alone can achieve successful results [2]. Empirical broad-spectrum intravenous antibiotics should be effective against common organisms that cause mastoiditis which include *Staphylococcus aureus, Streptococcus pneumoniae, Streptococcus pyogenes, Pseudomonas aeruginosa* and anaerobes [10, 11]. Delayed diagnosis and failure to respond to antibiotics may necessitate mastoidectomy and petrous apicectomy [12].

Our patient responded well to intravenous ceftazidime and vancomycin hence mastoidectomy deemed unnecessary. Tympanostomy and Grommet insertion was performed due to persistent middle ear effusion. Optimum duration of antibiotics is controversial but, usually ranges from 3 to 5 weeks [5]. Our patient received antibiotics for a total duration of 6 weeks.

In conclusion, this case report highlights the importance of a thorough physical examination in children presenting with unrelated neurological symptoms and signs. Abducens nerve palsy should raise the suspicion of raised intracranial pressure as well as local causes and neuroimaging is vital in situations of diagnostic uncertainty. Gradenigo syndrome emphasises the importance of incorporating anatomical knowledge into clinical practice and timely intervention, which will avoid the need for surgery as demonstrated in this case report.

## Data Availability

Not applicable

## Abbreviations

CSF: Cerebrospinal fluid
CT: Computed tomography
MRI: Magnetic Resonance Imaging
